# Genome-Wide Analysis Indicates a Complete Prostaglandin Pathway from Synthesis to Inactivation in Pacific White Shrimp, *Litopenaeus vannamei*

**DOI:** 10.3390/ijms23031654

**Published:** 2022-01-31

**Authors:** Hao Yang, Xiaoli Chen, Zhi Li, Xugan Wu, Mingyu Zhou, Xin Zhang, Yujie Liu, Yuying Sun, Chunhua Zhu, Qiuhui Guo, Ting Chen, Jiquan Zhang

**Affiliations:** 1 Laboratory of Zoological Systematics and Application of Hebei Province, College of Life Sciences, Hebei University, Baoding 071002, China; glitter_aug@163.com (H.Y.); yifu912lab@163.com (Y.L.); sunyuying125@163.com (Y.S.); 2 Guangdong Research Center on Reproductive Control and Breeding Technology of Indigenous Valuable Fish Species, Fisheries College, Guangdong Ocean University, Zhanjiang 524088, China; chenxl806@163.com (X.C.); zhu860025@163.com (C.Z.); 3 Key Laboratory of Exploration and Utilization of Aquatic Genetic Resources, College of Fisheries and Life Science, Shanghai Ocean University, Shanghai 201306, China; lizhilds009@163.com (Z.L.); xgwu@shou.edu.cn (X.W.); zmy9180@163.com (M.Z.); 4 CAS Key Laboratory of Tropical Marine Bio-Resources and Ecology (LMB), South China Sea Institute of Oceanology, Chinese Academy of Sciences, Guangzhou 510301, China; zhangxin174@mails.ucas.ac.cn; 5 EasyATGC Limited Liability Company, Shenzhen 518081, China; gqiuhui@163.com

**Keywords:** prostaglandin, synthesis and inactivation, genome-wide analysis, mRNA expression, crustacean

## Abstract

Prostaglandins (PGs) play many essential roles in the development, immunity, metabolism, and reproduction of animals. In vertebrates, arachidonic acid (ARA) is generally converted to prostaglandin G_2_ (PGG_2_) and H_2_ (PGH_2_) by cyclooxygenase (COX); then, various biologically active PGs are produced through different downstream prostaglandin synthases (PGSs), while PGs are inactivated by 15-hydroxyprostaglandin dehydrogenase (PGDH). However, there is very limited knowledge of the PG biochemical pathways in invertebrates, particularly for crustaceans. In this study, nine genes involved in the prostaglandin pathway, including a *COX*, seven *PGS*s (*PGES*, *PGES2*, *PGDS1/2*, *PGFS*, *AKR1C3*, and *TXA2S*), and a *PGDH* were identified based on the Pacific white shrimp (*Litopenaeus vannamei*) genome, indicating a more complete PG pathway from synthesis to inactivation in crustaceans than in insects and mollusks. The homologous genes are conserved in amino acid sequences and structural domains, similar to those of related species. The expression patterns of these genes were further analyzed in a variety of tissues and developmental processes by RNA sequencing and quantitative real-time PCR. The mRNA expression of *PGES* was relatively stable in various tissues, while other genes were specifically expressed in distant tissues. During embryo development to post-larvae, *COX*, *PGDS1*, *GDS2*, and *AKR1C3* expressions increased significantly, and increasing trends were also observed on *PGES*, *PGDS2*, and *AKR1C3* at the post-molting stage. During the ovarian maturation, decreasing trends were found on *PGES1*, *PGDS2*, and *PGDH* in the hepatopancreas, but all gene expressions remained relatively stable in ovaries. In conclusion, this study provides basic knowledge for the synthesis and inactivation pathway of PG in crustaceans, which may contribute to the understanding of their regulatory mechanism in ontogenetic development and reproduction.

## 1. Introduction

Prostaglandins (PGs) are an important class of eicosanoids, which are lipid signals derived from polyunsaturated fatty acids (PUFAs) [1,2,3,4]. In humans, PGs are produced in almost every tissue [5] as autocrine and paracrine regulators and may regulate many physiological processes within the central nervous system [6,7], gastrointestinal system [8,9], and cardiovascular system [10,11,12]. As in terrestrial vertebrates, prostaglandins in marine vertebrates are implicated in reproduction, cardiovascular systems, osmoregulation, and oxygenation regulation [13].

In humans, PGs are produced by the serial metabolic cascades of phospholipids cyclooxygenase (COX) and prostaglandin synthases (PGSs) and finally inactivated by 15-hydroxyprostaglandin dehydrogenase (PGDH). The synthesis and inactivation pathways of PGs are conserved in mammals but partially missing in other animal groups, such as fish [14] and insects [15,16]. However, little is known regarding the PG synthesis and inactivation pathways of crustaceans.

COX, also known as prostaglandin G/H synthase (PGHS) or prostaglandin endoperoxide H synthase (PGHS), is a glycoprotein with both peroxidase and PGS activities [17] that catalyzes the formation of PGs, thromboxanes, and levoglandins, including COX1 and COX2 [18]. In the prostaglandin synthesis pathway, COX can catalyze the formation of arachidonic acid first to PGG_2_ and subsequently to PGH_2_ [19,20]. PGH_2_ serves as a substrate for cell- and tissue-selective prostanoid synthases and isomerases that produce numerous bioactive prostanoids [21]. The exceptions are found in insects, in which ARA converts to PGH_2_ by the action of peroxidase rather than by COX [15,22].

During the synthesis of various downstream active prostaglandins, prostaglandin E synthases convert PGH_2_ to PGE_2_. Three types of PGES have been identified in vertebrates, including membrane-associated cytosolic prostaglandin E synthase 1 (PGES1), membrane-associated prostaglandin E synthase 2 (PGES2), and cytosolic prostaglandin E synthase (cPGES) [23,24]. Likewise, prostaglandin D synthases (PGDS) are classified into two types: lipocalin-type PGDS, which was previously known as the brain-type enzyme or glutathione (GSH)-independent enzyme, and hematopoietic PGDS, the spleen-type enzyme or GSH-requiring enzyme [25]. Prostaglandin F_2_ (PGF_2_) is synthesized with three substrates of PGE_2_, PGD_2_, or PGH_2_ through PGE 9-keto reductase, PGD 11-keto reductase (AKR1C3), and PGH 9,11-endoperoxide reductase (PGFS), respectively [26]. In addition, PGH_2_ is used as a substrate in the synthesis of prostaglandin I_2_ (PGI_2_) and thromboxane A_2_ (TxA_2_) by PGI synthase (PGIS) and TxA_2_ synthase (TxA_2_S), respectively [27,28]. TxA_2_ is an extremely unstable molecule and may rapidly convert to inactive thromboxane B_2_ (TxB_2_) [29].

Furthermore, PGs are primarily decomposed by the initial oxidation of 15 (S)-hydroxyl groups catalyzed by 15-hydroxyprostaglandin dehydrogenases (PGDHs) to 15-ketoprostaglandins (15-keto PGE_2_s), whose biological activity is significantly lower than that of PGs [30]. PGDH can use a wide range of prostaglandins as substrates, such as PGE_1_ and PGE_2_, but exclude PGI_2_, PGD_2_, and TXB_2_ [31].

In crustaceans, the processes of growth, development, and reproduction are delicately regulated by a variety of hormones [32,33,34,35,36,37]. Previous studies have indicated that PGs are involved in reproduction, including vitellogenesis and spawning in the giant river prawn *Macrobrachium rosenbergii* and the penaeid shrimp *Marsupenaeus japonicus* [38,39]. During the ovarian development of the penaeid shrimp *Penaeus japonicas*, particularly in the vitellogenesis stage, the amounts of PGE_2_ are increased [40]. In the crabs *Scylla olivacea, Scylla serrata,* and *Oziotelphusa senex*, PGs have been found to be regulators of molting and ovarian maturation [41,42]. However, studies regarding the whole PG pathway from synthesis to inactivation are still limited in crustaceans.

Pacific white shrimp (*Litopenaeus vannamei*) is the most economically important crustacean species in global aquaculture [43], and their ovaries are often unable to mature naturally but are induced to mature following artificial unilateral eyestalk ablation and nutritional supplementation [44]. The live polychaetes (*Perinereis* sp.) that contained PGE_2_ is one of the most effective nutritional supplementations for the oocyte development of penaeid shrimp, especially during late development and ovulation [45]. In the present study, we first identified nine genes in the PG synthesis and inactivation pathway based on the screening of the *L. vannamei* genome. These genes were analyzed with bioinformatics methods, and their expression patterns at the transcriptional level were confirmed by transcriptomic RNA sequencing (RNA-seq) and quantitative real-time PCR (qRT–PCR). Based on this study in a penaeid shrimp, we aimed to illustrate the specific synthesis and inactivation models of PG in crustaceans, which may contribute to understanding the potential regulatory mechanism of PGs in the development and reproduction of crustaceans.

## 2. Results

### 2.1. Screening of Genes in the Prostaglandin Pathway

**Figure 1:**
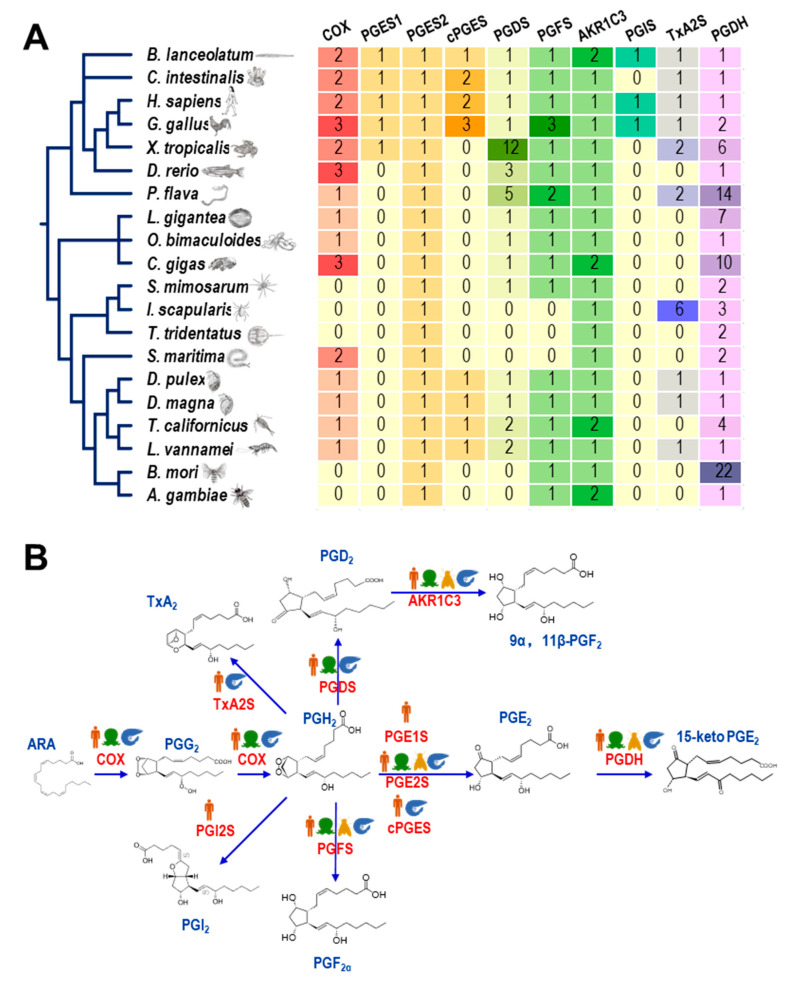
(**A**) Gene numbers of COX, PGSs, and PGDH in 20 species from different taxa; (**B**) presences of genes involved in the prostaglandin pathway from four major taxa.

As shown in Figure 1A, the gene numbers of *COX*, *PGS*s, and *PGDH* varied among animal species of different taxa. The *COX* genes were widely present in all species of the Deuterostomia and Lophotrochozoa but lost in some taxa of the Ecdysozoa. In Arthropoda, *COX* genes can only be found in Crustacea (e.g., *Daphnia pulex*, *Daphnia magna*, *Tigriopus californicus,* and *L. vannamei*) and Myriapoda (e.g., *Strigamia maritima*) but not in Insecta (e.g., *Bombyx mori* and *Anopheeles gambiae*), Arachnoidea (e.g., *Stegodyphus mimosarum* and *Ixodes scapularis)* or Merostomata (e.g., *Tachypleus tridentatus*). For the three types of *PGES*, *PGES1*s were rarely identified in vertebrates, *PGES2*s were observed in all 20 species, and *cPGES*s were present in vertebrates and crustaceans. *PGDS*, *PGFS*, and *AKR1C3* were also found in crustaceans. However, *PGIS* was present in some vertebrate species. On the other hand, *PGIS* was limited in some Deuterostomia species. TxA2Ss were widely found in Deuterostomia but lost in Lophotrochozoa, while they were present in some Ecdysozoa arthropod species, including *L. vannamei*. Furthermore, *PGDH*s were found in all species we analyzed, while their gene numbers were expanded independently in some species, such as *Ptychodera flava*, *Crassostrea gigas*, and *B. mori*, but retained a single copy in *L. vannamei*.

The synthesis and inactivation pathways of PGs in the four major taxa (mammals, mollusks, insects, and crustaceans) are illustrated in Figure 1B. In total, nine genes involved in the PG pathway were present in *L. vannamei* (Table 1), as a representative crustacean, while *PGIS* genes were absent, indicating a relatively complete PG synthesis and inactivation pathway in shrimp compared to mammals.

### 2.2. Comparative Analysis of COX in L. vannamei and Other Species

**Figure 2:**
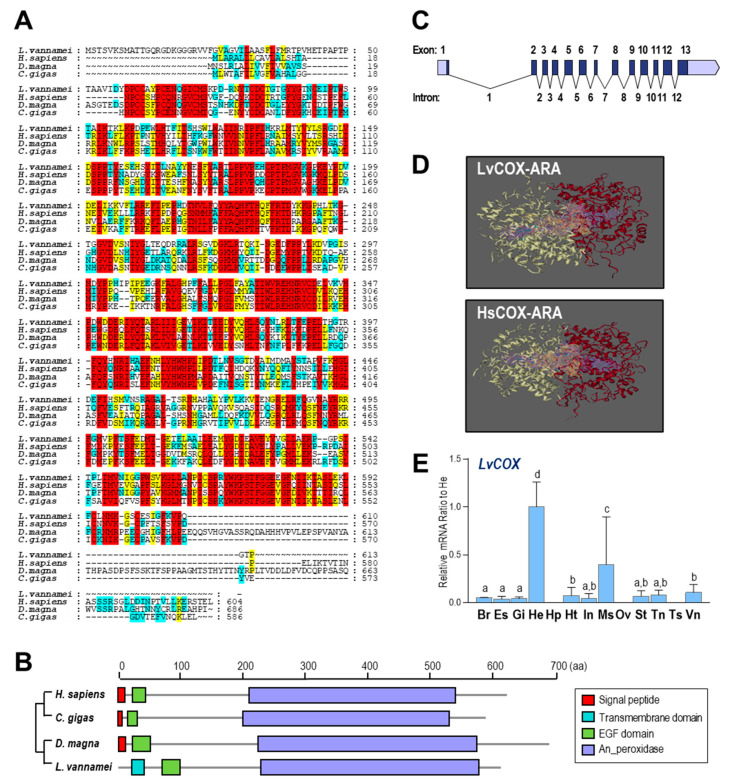
(**A**) Alignment of the COX a.a. sequences among four species including *L. vannamei* (XP027218437.1), *H. sapiens* (NP000954), *D. magna* (XP032795448.2), and *C. gigas* (XP011440168.2). The conserved residues are boxed in red, and similar residues are labeled in yellow; (**B**) the structural domains of COX proteins predicted by SMART; (**C**) exon/intron organization of *LvCOX* gene. The ORFs and UTRs in exons are marked in dark gray and empty boxes, respectively, and the introns are labeled with solid lines. The positions for 13 exons are 231–283, 2444–2557, 2723–2842, 2971–3114, 3307–3450, 3617–3798, 4019–4102, 4493–4633, 4943–5057, 5228–5403, 5561–5672, 5825–6038 and 6196–6444, and for 12 introns are 289–2443, 2558–2722, 2843–2970, 3115–3306, 3451–3616, 3799–4018, 4103–4492, 4634–4942, 5058–5227, 5404–5560, 5673–5824, and 6039–6195; (**D**) 3D structures and substrate binding regions of COXs in *L. vannamei* and *H. sapiens*; (**E**) relative mRNA ratio of *LvCOX* transcripts among different tissues including the brain (Br), eyestalk (Es), gills (Gi), hemocyte (He), hepatopancreas (Hp), heart (Ht), intestines (In), muscle (Ms), ovary (Ov), stomach (St), thoracic ganglion (Tn), testis (Ts), ventral ganglion (Vn). The experimental groups denoted by the same letter represent a similar level of transcript expression (*p* > 0.05, ANOVA, followed by Fisher LSD test).

Multiple alignments for COX amino acid (a.a.) sequences are shown in Figure 2A. COX proteins from four species (*Homo sapiens*, *C.* *gigas*, *D.* *magna*, and *L.* *vannamei*) showed considerable consensus sequences, indicating the conservation of these endoperoxidases. Based on the SMART program, the transmembrane domain, epidermal growth factor (EGF) domain, and animal haem peroxidase (An_peroxidase) domain were found in the a.a. sequence of LvCOX. However, for COXs from *H. sapiens*, *M.* *musculus*, and *Danio rerio*, signal peptides were found instead of the transmembrane domain in LvCOX (Figure 2B), suggesting a possible working model for COX in the shrimp.

The gene sequence of *LvCOX* was obtained and deposited in GenBank under accession no. LOC113810970. The *LvCOX* gene is 7268 bp in length, containing 13 exons separated by 12 introns. All 12 introns begin with GT and end with AG, conforming to the GT/AG rule of intron splicing (Figure 2C). By using the SWISS-MODEL program, the three-dimensional (3D) structures of COX from *H.* *sapiens* and *L.* *vannamei* were constructed for comparison. In this case, the binding regions of HsCOX and LvCOX were similar in their 3D structures. Each COX protein was composed of two subunits that formed a binding pocket between them, as shown by the purple region in Figure 2D, which can combine ARA and bind in the yellow region.

The mRNA expression profile of *LvCOX* among different tissues was analyzed by qRT–PCR. As shown in Figure 2E, *LvCOX* was highly expressed in the hemocytes followed by the muscle and showed much lower expression in other tissues. However, no detectable expression of *LvCOX* was found in the hepatopancreas and reproductive organs—namely, the ovary and testis.

### 2.3. Comparative Analysis of PGSs in L. vannamei and Other Species

**Figure 3:**
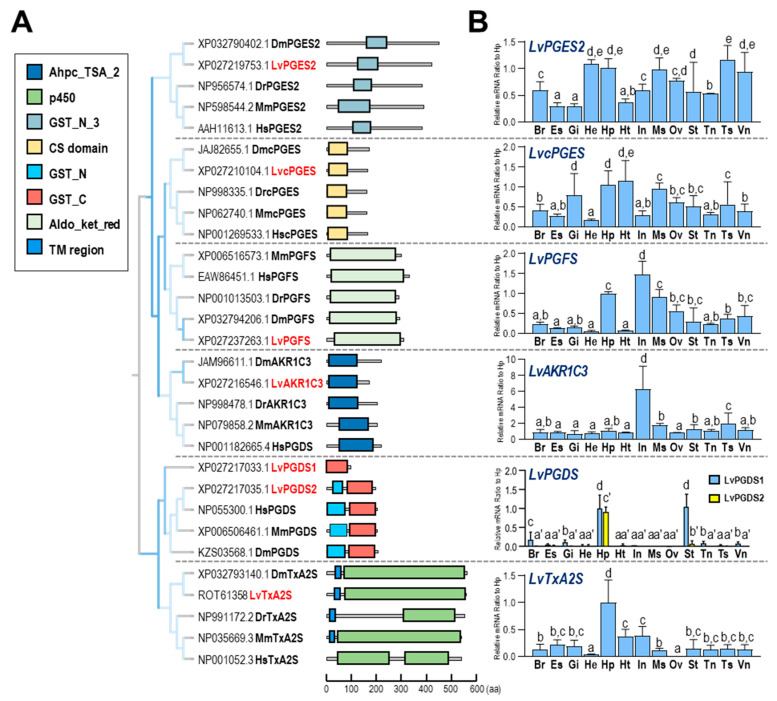
(**A**) phylogenetic analysis and the structural domain organization of multiple *PGS* genes in various species. The selected species include *D. magna*, *L. vannamei*, *H. sapiens*, *D. rerio*, and *Mus musculus*; (**B**) relative mRNA ratio of multiple *LvPGS* genes (*LvPGES2*, *LvcPGES*, *LvPGFS*, *LvAKR1C3*, *LvPGDS1*, *LvPGDS2*, and *LvTxA2S*) among different tissues, including brain (Br), eyestalk (Es), gills (Gi), hemocyte (He), hepatopancreas (Hp), heart (Ht), intestines (In), muscle (Ms), ovary (Ov), stomach (St), thoracic ganglion (Tn), testis (Ts), and ventral ganglion (Vn). The experimental groups denoted by the same letter represent a similar level of transcript expression (*p* > 0.05, ANOVA, followed by Fisher LSD test).

In *L. vannamei*, two *PGES* genes were identified, corresponding to two types of *PGES*—namely, LvPGES2 with the GST_N_3 domain and LvcPGES with the CS domain, both of which were conserved with *PGES*s in other species. In addition, PGFS, AKR1C3, and TxA2S from different species shared high conservation within their functional domains, with an exception of human TxA2S, which does not contain a transmembrane region. Furthermore, the LvPGDS1 was not conserved with its counterparts in other species since it lacked the GST_N domain (Figure 3A).

The genes for different types of *PGES*s were expressed in distant patterns in selected tissues (Figure 3B). In this case, *LvPGES*2 was primarily expressed in the testis, hemocyte, hepatopancreas, and ventral ganglion, and *LvcPGES* was predominantly expressed in the heart, hepatopancreas, and gills. In addition, high expression of both *LvPGFS* and *LvAKR1C3* transcripts was observed in the intestines, and *LvTxA2S* expression was abundant in the hepatopancreas. Moreover, the expression profiles of *LvPGDS1* and *LvPGDS2* were distinct, in which the former was highly expressed in the hepatopancreas and stomach, and the latter was restrictedly expressed in the hepatopancreas.

### 2.4. Comparative Analysis of PGDH in L. vannamei and Other Species

**Figure 4:**
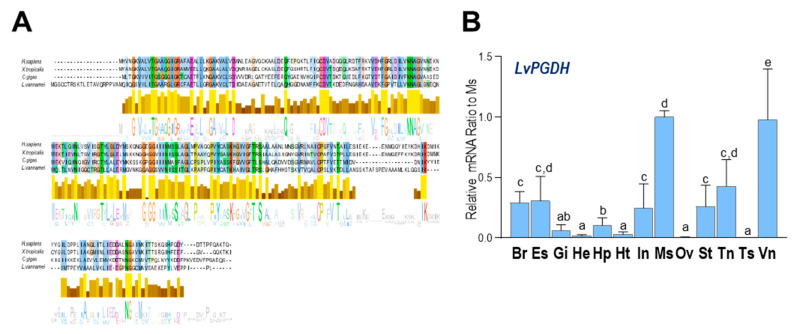
(**A**) Alignment of PGDH a.a. sequences among four species (*H. sapiens*, *X. tropicalis*, *C. gigas*, and *L. vannamei*); (**B**) relative mRNA ratio of *LvPGDH* transcripts among different tissues including the brain (Br), eyestalk (Es), gills (Gi), hemocyte (He), hepatopancreas (Hp), heart (Ht), intestines (In), muscle (Ms), ovary (Ov), stomach (St), thoracic ganglion (Tn), testis (Ts), ventral ganglion (Vn). The experimental groups denoted by the same letter represent a similar level of transcript expression (*p* > 0.05, ANOVA, followed by Fisher LSD test).

As shown in Figure 4A, multiple alignments of a.a. sequences indicated that the PGDH shared considerable consensus sequences between *H. sapiens*, *Xenopus tropicalis*, *C. gigas*, and *L. vannamei*, indicating a conserved inactivation pathway of PGs among diversified animal species. In addition, *LvPGDH* transcripts were found to be ubiquitously expressed in all examined tissues, and the highest expression was found in the muscle, followed by the ventral ganglion.

### 2.5. Expression Patterns during Ontogenetic Development, Ovarian Development, and Molting

**Figure 5:**
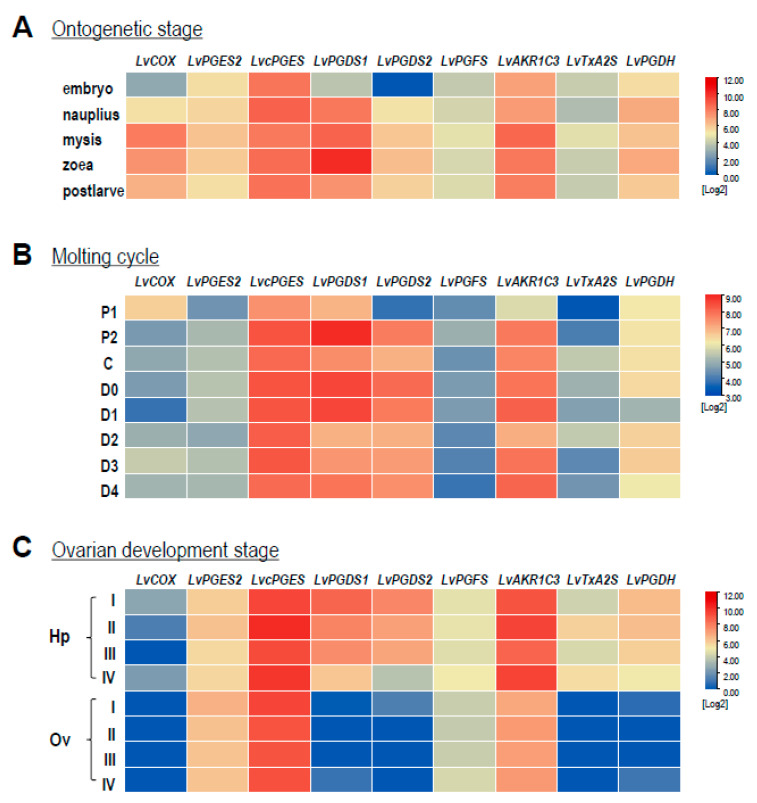
The expression profiles of genes involved in the PG pathway of *L. vannamei* during different developmental processes: (**A**) ontogenetic stages, including embryo, nauplius, mysis, zoea, and post-larvae; (**B**) molting cycle, including postmolt (P1 and P2 stages), intermolt (C stage) and premolt (D0, D1, D2, D3, and D4 stages); (**C**) ovarian development, including hepatopancreas (Hp) and ovaries (Ov) in stages I to IV.

Based on the RNA-seq data, transcript levels of the *COX*, *PGS*s, and *PGDH* in *L. vannamei* were analyzed during the developmental processes of ontogenetic stages, ovarian development, and the molting cycle. As shown in Figure 5A, the expression patterns of *LvPGES2*, *LvPEGS*, *LvPGFS*, *LvTxA2S*, and *LvPGDH* were almost identical and did not change noticeably at all stages during ontogenesis, except that the expression level of *LvPGES2* was relatively higher than other genes. On the other hand, the expression of the remaining genes showed dynamic changes, e.g., *LvCOX*, *LvPGDS2*, and *LvAKR1C3* showed their highest expression levels at the mysis stage than at other stages, while the highest expression of *LvPGDS1* exhibited at the zoea stage. During the molting cycle (Figure 5B), the P1 stage was a transition point for the expression of most genes in the PG-related pathways. In this case, the transcript levels of *LvPGES2*, *LvcPGES*, *LvPGDS1*, *LvPGDS2*, *LvAKR1C3*, and *LvTxA2S* were all reduced at the P1 stage, while *LvCOX* showed the highest expression level at this stage. In addition, significantly low expression of *LvCOX* and *LvPGDH* appeared at the D1 stage, and the expression of *LvPGFS* did not change markedly at any stage. During ovarian development (Figure 5C), *LvCOX*, *LvPGES2*, and *LvcPGES* were stably expressed in both the hepatopancreas and ovary, while the *LvPGDS1*, *LvPGDS2*, *LvPGFS*, *LvTxA2S*, and *LvPGDH* expression was higher in the hepatopancreas than in the ovary.

## 3. Discussion

In this study, enzymes related to the prostaglandin pathway were screened based on the *L. vannamei* genomic data. The identified enzymes can support a complete PG synthesis pathway from ARA to PGG_2_ (Figure 1B), along with PGH_2_ to various PGs, followed by the presence of an inactivation pathway. By analysis of functional domains (Figure 2B, Figure 3A, and Figure 4A), most of the enzymes in the PG pathway of *L. vannamei* show strong conservation with their counterparts in other species, indicating that it is an ancient pathway in metazoans. In addition, the expression of these genes was determined in various tissues and during multiple physiological processes. This evidence will facilitate further studies regarding the specific functions of these enzymes in crustaceans and the PG-mediated applications in shrimp culture.

In most species, COX1 and COX2 are considered constitutive and inducible COXs, respectively [46]. Both enzymes catalyze a cyclooxygenase reaction in which ARA is converted to PGG_2_ and a peroxidase reaction in which PGG_2_ is reduced to PGH_2_. A previous study showed that the major sequence differences between COX isoforms occur in the signal peptide domains [47]. In this study, however, only a single *LvCOX* gene was identified from the *L. vannamei* genome, and the N terminus of its protein product is a transmembrane structural domain instead of a signal peptide. Since their main functional domains are highly conserved, *Lv*COX and *Hs*COX are speculated to be essentially identical in terms of spatial structure and binding regions. In mammals, *COX1* is uniquely expressed in endothelial cells, but *COX2* is specifically expressed in some tissues (e.g., epithelial cells and muscle) after stimulation in various ways [48,49,50]. Similarly, *LvCOX* was highly expressed in the hemocytes and muscle, indicating the functional conservation of these enzymes among distant species. It is very interesting that the expression of *LvCOX* was absent in the hepatopancreas, ovary, and testis (Figure 2E). However, the effects of PGs on reproduction have been reported in several crustacean species [38,39,40,41,42], and the hepatopancreas and ovary are the major organs for vitellogenesis in *L. vannamei* [51]. It is logical to speculate that the PGs acting on the hepatopancreas and ovary are conversed by the exogenous intermediate PGH_2_.

PGES can isomerize PGH_2_ to PGE_2_, and molecular identification of multiple PGES enzymes has revealed the existence of two distant pathways for the biosynthesis of PGE_2_—namely, the constitutive COX1-cPGES pathway and the inducible COX2-mPGES pathway [52,53]. Both mPGES and cPGES were identified in shrimp, and their differences and specificity still need to be investigated. In addition, *cPGES* is most abundantly expressed in the testes of rats [53], identical to our current study, in which *LvcPGES* was expressed in the testis at the highest level.

In mammals, two PGDSs can isomerize PGH_2_ to PGD_2_—namely, lipocalin-type PGD synthase (L-PGDS) [54] and hematopoietic PGDS (H-PGDS) [55,56]. H-PGDS is a 26 kDa cytosolic protein responsible for the biosynthesis of PGD_2_ in immune and inflammatory cells, such as mast cells [57]. In *L. vannamei*, both *LvPGDS1* and *LvPGDS2* identified were related to human *H-PGDS*, and they were predominantly expressed in the hepatopancreas, which may respond to pathogenic infection in shrimp. However, *L-PGDS* has not been found in crustaceans, indicating that it is a specific isoform in vertebrates. PGDH catalyzes the reversible oxidation of the 15-hydroxyl group of prostaglandins to produce a 15-keto metabolite with greatly reduced biological activity [58]. Six a.a. residues (Gly, Gly, Gly, Asp, Tyr, and Lys) in PGDH are strictly conserved in the protein sequences from different species [59]. As shown in Figure 4A, although the LvPGDH a.a. sequences are longer than other species, they are conserved at these key positions for enzyme activities. In addition, the extended sequence in the N terminus of LvPGDH is not a signal peptide.

PGs are widely found in all tissues and body fluids in most metazoan animals. The synthesis and inactivation pathways of PG have been studied extensively in higher-order mammals but are still limited in low-order nonmammalian or invertebrate animals, such as crustaceans. In aquatic animals, studies regarding the function of PGs are mainly focused on ovarian development [60,61]. With its collective findings, this study provides new insights into the synthesis and inactivation pathway of prostaglandins in crustaceans and may contribute to their regulatory mechanism in development and reproduction; nevertheless, further studies need to be conducted to understand the functions and regulatory mechanism of these key genes and PGs in crustaceans in depth.

## 4. Materials and Methods

### 4.1. Cross-Genomic Analysis of Genes in the Prostaglandin Pathway

For cross-genomic analysis to identify genes in the prostaglandin pathway, the selected species included *Branchiostoma lanceolatum* (GCA_900088365.1), *D.* *magna* (GCA_003990815.1), *D. pulex* (GCA_911175335.1), *T. californicus* (GCA_007210705.1), *L. vannamei* (GCA_003789085.1), *A. gambiae* (GCA_000005575.1), *B. mori* (GCA_014905235.2), *I. scapularis* (GCA_016920785.2), *S. mimosarum* (GCA_000611955.2), *T. tridentatus* (GCA_004210375.1), *S. maritima* (GCA_000239455.1), *Octopus bimaculoides* (GCA_001194135.1), *C. gigas* (GCA_902806645.1), *Lottia gigantea* (GCA_000327385.1), *H. sapiens* (GCA_000001405.28), *Gallus gallus* (GCA_016699485.1), *X. tropicalis* (GCA_000004195.4), *D. rerio* (GCA_000002035.4), *Ciona intestinalis* (GCA_000224145.2), and *P. flava* (GCA_001465055.1). The gene functions were annotated based on the best-matched hits to SwissProt [62] (UniProt < EMBL-EBI, accessed on 21 July 2020) using BLAST 2.9.0+ [63] (-p blastp -e 1e-5) (BLAST: Basic Local Alignment Search Tool (nih.gov), accessed on 21 July 2020). The gene motifs and domains were identified from the InterPro member databases using InterProScanm [64] (v5.27-66.0) (InterPro (Ebi. ac. the UK), accessed on 21 July 2020) with parameters (-appl ProDom, PRINTS, Pfam, SMART, PANTHER, ProSiteProfiles, and ProSitePatterns). Related genes were identified by searching gene and domain description with keywords (such as cyclooxygenase) and manually checked considering phylogeny.

### 4.2. Phylogenetic Tree and Sequence Analysis

The evolutionary tree of 20 species was constructed through a Lifemap (Lifemap NCBI (univ-lyon1.fr), accessed on 16 August 2021). Alignment of COX, PGSs, and PGDH among various species was performed with the ClustalX program and demonstrated using ESPript 3.0 (ESPript 3.x/ENDscript 2.x (ibcp.fr), accessed on 16 August 2021) and the Genedoc program. The sequences were obtained from GenBank. The phylogenetic tree was constructed based on a.a. (*p* distance) with the neighbor-joining method (pairwise deletion) with 1000 bootstrap replicates using MEGA X. In this case, different PGS genes were constructed into a phylogenetic tree. The tree was visualized via iTOL (iTOL: Interactive Tree of Life (embl.de), accessed on 16 August 2021) and Evolview (EvolView: login (evolgenius.info), accessed on 16 August 2021).

### 4.3. Structural Analysis of Proteins

The gene structure for *LvCOX* was analyzed by mapping with Exon–Intron Graphic Maker (WormWeb.org—Exon–Intron Graphic Maker, accessed on 20 August 2021). The structural domains for COX and PGSs were predicted by using SMART (SMART: Main page (embl-heidelberg.de), accessed on 20 August 2021) and annotated by IBS (IBS—Online (biocuckoo.org), accessed on 20 August 2021). The three-dimensional (3D) models for COX were deduced with the knowledge-based modeling program ProMod II provided by the SWISS-MODEL server and were measured by using SAVES v6.0 (SAVESv6.0—Structure Validation Server (ucla.edu), accessed on 20 August 2021). The 3D chemical structures of ARA and PGs were downloaded from PubChem (PubChem (nih.gov), accessed on 20 August 2021). The ARA binding sites and modes for COXs were predicted by using Proteins Plus (Zentrum für Bioinformatik: Universität Hamburg—Proteins Plus Server, accessed on 20 August 2021).

### 4.4. Animals and Tissue Samples Collection

Adult Pacific white shrimp were collected from the Jinyang Shrimp Culture Center, Maoming, Guangdong, China, and maintained in artificial seawater (30 parts per thousand ppt and pH 8.2) at 28 °C under a 12 h dark–12 h light photoperiod. Six individuals of shrimp were dissected directly on ice after being collected from the pond. In total, 13 tissues were harvested, including the brain, eyestalk, gills, hemocytes, hepatopancreas, heart, intestines, muscle, ovary, stomach, thoracic ganglion, testis, and ventral ganglion. In this case, ovary and testis samples were harvested from sexually mature shrimp, and other samples were collected from sexually immature shrimp. Samples were immediately frozen in liquid nitrogen and stored at −80 °C for further mRNA experiments. All efforts were made to minimize animal suffering. This article does not contain any studies with human participants.

### 4.5. Realtime PCR for Tissue Distribution

For tissue distribution, total RNA was isolated using RNAiso Plus (Invitrogen, Carlsbad, CA, USA), digested with gDNA Eraser (TaKaRa, Dalian, China), and reverse transcribed using the PrimeScript RT reagent Kit (TaKaRa). The first-strand cDNA samples obtained were then subjected to qPT–PCR using a RotorGene RG-3000 real-time PCR system (Corbett Research, San Francisco, CA, USA). PCRs were conducted using SYBR Premix Ex Taq II (TaKaRa) with specific primers for their target genes (Table 2). The PCR cycle numbers were fixed to 40 with 30 s at 94 °C for denaturing, 30 s at 60 °C for annealing, 30 s at 72 °C for extension, and 20 s at 82 °C for signal capture. In this case, the transcript expression of the target genes was routinely normalized using *β-actin* mRNA. The relative expression levels were calculated using the comparative Ct method with the 2^−^^ΔΔCt^ formula [65]. The raw data for the expression of different genes were transformed as ratios of control groups (e.g., hepatopancreas, hemocyte, or muscle). The data expressed as the mean ± standard error (SE) were analyzed by using one-way ANOVA, followed by Fisher’s least significant difference (LSD) test.

### 4.6. Transcriptomic Analysis for Gene Expression

Transcript levels of prostaglandin-pathway-involved genes in *L. vannamei* during different developmental processes were further analyzed with transcriptomes. The transcriptomic data for ontogenetic development stages (PRJNA253518) [66] and molting cycle (PRJNA288849) [67] were obtained from GenBank, and those for ovarian development were previously established by our laboratory (SUB10677615). For the transcriptome of ontogenetic development, the embryo (E) sample was from an equivalent RNA mixture of the zygote, blastula, gastrula, limb bud embryo, and larva in membrane stages; the nauplius (N) sample was from an equivalent RNA mixture of nauplius I, III, and VI stages; the zoea (Z) sample was from an equivalent RNA mixture of zoea I, II, and III stages; the mysis (M) sample was from an equivalent RNA mixture of mysis I, II, and III stages; RNA from post-larvae 1 was considered as the post-larvae (P) sample. For the transcriptome of the molting cycle, the molting stages were classified as postmolt (P1 and P2), inter-molt (C), and pre-molt (D0, D1, D2, D3, and D4). For the transcriptome of ovarian development, the stages were clarified into I–IV, based on the classification of predominant oocytes, as described previously [51]. A matrix of read counts mapped to *L. vannamei* genomic features (genes) was extracted directly from the files generated by StringTie (run with the -e parameter) with script prepDE.py. The read count per million reads (CPM) was calculated, and cross-sample normalization was performed using edgeR [68] and DESeq2 [69]. The obtained heatmaps were then created and clustered with hierarchical clustering using Tb tools (Guangzhou, China).

## Figures and Tables

**Table 1:** Genes involved in the PG pathway in *L. vannamei*.

Protein ID	Annotation
XP027210685.1	Phospholipase A2
XP027215735.1	Phospholipase A2
XP027216925.1	Phospholipase A2
XP027224729.1	Phospholipase A2
XP027229029.1	Phospholipase A2
XP027230031.1	Phospholipase A2
XP027239642.1	Phospholipase A2
XP027218437.1	Cyclooxygenase/Prostaglandin G/H synthase
XP027219753.1	Prostaglandin E synthase
XP027224753.1	Prostaglandin E synthase
XP027210104.1	Prostaglandin E synthase
XP027217033.1	prostaglandin D synthase
XP027217035.1	prostaglandin D synthase
XP027216546.1	Prostamide/prostaglandin F synthase
XP027237263.1	9,11-endoperoxide prostaglandin H2 reductase

**Table 2:** Primers sequences used in this study.

Gene Name.	Forward Primer (5′-3′)	Reverse Primer (5′-3′)	Product (bp)
*LvCOX*	ATGTCAACGTCTGTGAAAAGCATGG	CCATGCTTTTCACAGACGTTGACAT	174
*LvPGES2*	CTCTGAGGGCAGCGAGTA	GCAACAGAATGGGCAAGT	133
*LvPGES3*	CAGCACTGGCTCAAAGTA	GTCGGAATCTTCATCCTC	168
*LvPGDS1*	TGGCAGACAAGGACGAGG	GCCGAGGCTGATGATGAA	162
*LvPGDS2*	GATGTCTTGGGTCGATCTGA	AAGGTGTCTGGGCGTCTTT	159
*LvPGFS*	CGGAATGAAACTGAAATAGG	GAGGTAGGTCAGCTGCAGGTTACTT	178
*LvAKR1C3*	GAGGCTCATTGGCATCGG	AATCATCGCAAGAACACC	145
*LvTxA2S1*	CGGAATGAAACTGAAATAGG	GGTAAAGGTCGAGGTAGGT	178
*LvPGDH*	CTGACCTCCTCGCCAACTCC	AGGGCTCTTTCTCCGCCTC	180

## Data Availability

The data presented in this study are available in the article.

## Abbreviations

COX	cyclooxygenase
PGES	prostaglandin E synthase
PGDS	prostaglandin D synthase
PGFS	prostaglandin F synthase/PGH 9,11-endoperoxide reductase
PGIS	prostaglandin I synthase
AKR1C3	PGD 11-keto reductase
TxA2S	thromboxane A2 synthase
PGDH	15-hydroxyprostaglandin dehydrogenases
